# HIV-1 Tat protein alter the tight junction integrity and function of retinal pigment epithelium: an *in vitro *study

**DOI:** 10.1186/1471-2334-8-77

**Published:** 2008-06-06

**Authors:** Ling Bai, Zhenping Zhang, Hui Zhang, Xiumei Li, Qiurong Yu, Haotian Lin, Wenhui Yang

**Affiliations:** 1 Key Laboratory of Ophthalmology, Ministry of Education; Zhongshan Ophthalmic Center, Sun Yat-sen University, Guangzhou 510060, PR China; 2 Department of Medicine, Division of Infectious Diseases, Center for Human Virology, Thomas Jefferson University, Philadelphia, USA

## Abstract

**Background:**

How HIV-1 enter into the eyes remains obscure. We postulated that HIV-1 Tat protein can alter the expression of specific tight-junction proteins and disturb the blood retinal barrier, and contributes to HIV trafficking into the eyes. This study is to determine the effects of HIV-1 Tat proteins on the barrier function and tight-junction protein expression of retinal pigment epithelial cell (RPE).

**Methods:**

A human RPE cell line (D407) cultured on microporous filter-supports was used. After treating with HIV-1 Tat protein, transepithelial electrical resistance (TER) of confluent RPE cells was measured by epithelial voltmeter. The permeability of the RPE cells to sodium fluorescein was measured. The expressions of the occludin and claudins were determined by real-time polymerase chain reaction, immunofluorescence, and Western blot analysis. Activation of ERK1/2 was detected by Western blot analysis with specific antiphospho protein antibodies. NF-κB DNA binding activity was determined by transcription factor assay. Specific pharmacologic inhibitors directed against the MAPKs were used to analyze the signaling involved in barrier destruction of RPE cells exposed to HIV-1 Tat.

**Results:**

Treating cultured human retinal pigment epithelial cells with 100 nM Tat for 24 hours increased the permeability and decreased the TER of the epithelial monolayer. HIV-1 Tat also disrupted and downregulated the tight-junction proteins claudin-1, claudin-3, and claudin-4 in these cells, whereas claudin-2 was upregulated, and the expression of occludin was unaffected. HIV-1 Tat protein also induced activation of ERK1/2 and NF-κB. HIV-1 Tat protein induced barrier destruction, changes in expression of TJs, and activation of ERK1/2 and NF-κB were abrogated by inhibitor of ERK1/2 and NF-κB.

**Conclusion:**

HIV-1 Tat protein causes increases in the paracellular permeability of RPE cells in vitro concomitant with changes in expression of certain transmembrane proteins associated with the tight junction. The effects of HIV-1 Tat on barrier function of the RPE may be mediated by ERK MAPK and NF-κB activation, which may represent potential targets for novel therapeutic approaches for the retinopathy induced by HIV infection.

## Background

Serious ophthalmic diseases can cause blindness in the absence of prompt diagnosis and therapy. These diseases often result from opportunistic infections and are common in HIV-infected patients [1]. The exact mechanism underlying the HIV invasion of ocular tissues is still poorly understood.

HIV-1 transactivator Tat protein (HIV-1 Tat) plays a pivotal role in both the HIV-1 replication cycle and the pathogenesis of HIV-1 infection. HIV-1 Tat modulates the expression of several cellular genes and triggers the activation of certain signal transduction pathways and transcription factors, suggesting a complex role in HIV-1 infection [2-5]. Extensive data document the pleiotropic effects of Tat protein in many host cells, particularly in cells targeted by HIV, and these effects induce the appearance of many systemic complications of AIDS, such as, HIV-associated dementia, HIV-associated nephropathy, and HIV-associated adipose redistribution syndrome [6-8]. Despite the importance of HIV-1 Tat, few reports have examined its potential role in HIV-associated ocular diseases [1,9].

The retinal pigment epithelium (RPE) lies between the photoreceptors of the neurosensory retina and the choroidal capillary bed, and depends on tight junctions (TJs) to forms a highly selective and regulateable barrier between the retina and choroid, called the outer blood-retina barrier (oBRB), that is responsible for the transport of nutrients and ions between photoreceptors and the choriocapillaris, and is very important for maintaining the normal vision [10]. The TJ, which is the most apical component of the junctional complex, represents the anatomic substrate of the oBRB. The composition of TJs, which has been unraveled over the past few years, is dominated by two main transmembrane proteins, occludin and claudins, which appear to be important to the tissue- and cell-specific function of TJs [11,12].

HIV-1 Tat protein can alter the expression of specific TJ proteins in brain microvascular endothelial cells (BMECs), which disturb the blood-brain barrier (BBB) and contributes to HIV trafficking into the brain [13,14]. Recently, it was demonstrated that the transport and permeation characteristics of BBB and oBRB, which is formed by the intercellular TJs of the RPE, are surprisingly similar [15]. The RPE is also one of the cells targeted by HIV, and the junctional integrity of the RPE can be affected by many factors [16-20]. We therefore hypothesized that HIV-1 Tat can alter the protein expression of TJs in the RPE, and thereby disturb the barrier function of oBRB, which may be one of the mechanisms for HIV-1 entry into the eyes.

The objectives of the present study were (1) to characterize the effects of HIV-1 Tat protein on the barrier function of cultured RPE cells, through transepithelial electrical resistance (TER) and permeability to fluorescence sodium, (2) to determine the differential regulation of transmembrane protein expression associated with the changes in barrier function, and (3) to determine the intracellular pathways that participate in changes in RPE induced by HIV-1 Tat.

## Methods

### Reagent

Dulbecco's modified Eagle's medium/High Glucose (DMEM), fetal bovine serum (FBS), penicillin and streptomycin were purchased from Hyclone (Logan, UT). Rabbit anti-occludin, claudin-1, claudin-2, and claudin-3, and mouse anti-claudin-4 were obtained from Zymed Laboratories (San Francisco, CA). The monoclonal antibody (mAb) to phospho-ERK was purchased from Cell Signaling Technology (Beverly, MA). The rabbit anti-ERK, used as controls for equal loading, was obtained from Santa Cruz Biotechnology (Santa Cruz, CA). Goat anti-rabbit and mouse IgG with a FITC conjugate were obtained from Sigma (St. Louis, MO). PD98059 was purchased from Calbiochem (San Diego, CA) and made up with dimethyl sulfoxide (DMSO) at 1 mM stock solution. Pyrrolidien dithiocarbamate (PDTC) was purchased from Sigma and dissolved in PBS. NE-PER^® ^Nuclear and Cytoplasmic Extraction Reagents was purchased from Pierce (Rockford, IL). Sodium fluorescein (MW: 376 Da) was purchased from Amersco (Solon, OH).

### Cell culture

The human RPE D407 cell line was generously provided by Dr Guo Zhongmin (Center of Experimental Animal Sun Yat-sen University). Cells were cultured in DMEM with high glucose (4.5 g/l), containing 10% FBS, penicillin (100 U/ml) and streptomycin (100 U/ml). The medium was changed every 2 days, and cells were subcultured by trypsinization every 4 days at a split of 1:5.

### Tat protein preparation and treatment

The 86-amino acid isoform of the Tat protein was obtained from The National Institutes of Health AIDS Reagent Program (Rockville, MD). It was reconstituted in phosphate-buffered saline (PBS) containing 1 mg/ml bovine serum albumin (BSA) and 0.1 mM dithiothreitol and deaerated by bubbling with helium. The protein was stored at -80°C in the dark before use. The specificity of Tat-mediated effects was assessed by treating cells with heat inactivated Tat prepared by incubating the protein at over 85°C for 30 min. Because Tat binds strongly to serum proteins, all experiments were carried out in serum-free media. D407 cells remained healthy and viable under these experimental conditions. The Tat treatment in the present study involved exposing D407 cells exposure to 100 nM Tat for 24 hours, which has frequently been used in previous in vitro studies [21,22].

### Cell viability assay

Cells were grown in 96-well plates at a density of 1 × 10^4 ^cells/well. After the indicated treatments, MTT(3-[4,5-dimethylthiozol-2-yl]-2,5-diphenyl tetrazoliumbromide) was added at 5 mg/ml to each well for 4 hours, after which the culture medium was removed and 150 μl of DMSO was added to each well. The absorbance was measured at 490 nm using a multifunctional microplate reader (POLARstar, OPTIMA, Germany).

### Measurement of TER

Transparent Millicell-CM filters (diameter of 12 mm, pore size of 0.4 μm, effective membrane area 0.6 cm^2^, membrane material: hydrophilic PTFE (Millipore, Bedford, MA) were coated with 50 μl of a rat-tail collagen I/ethanol mixture (Sigma) and left to dry before cells were subcultured. D407 cells were seeded at a density of 10^4 ^cells/filter on the filters was supported by 24-well culture plates. The volumes on the apical and basolateral side (inside and outside of the membrane) were 400 μl and 600 μl, respectively. The fluid pressure was the same in the two chambers.

The cultures were incubated in a humidified atmosphere (37°C, 5% CO_2_). The medium was changed on the following day, and subsequently changed every second day for the duration of the experiment. Phase contrast microscopy revealed that cells reached confluence at day 3, and then serum concentration of the culture medium was reduced to 1%. From 2 days after seeding, the TER was measured by an epithelial voltohmeter (EVOM, World Percision Instruments, USA) every other day to monitor the time course of the TER. We began the indicated treatments at day 10, the culture medium in control group also changed into serum free, and measured the TER at 1, 2, 3, 12, 24, 48, and 72 hours after exposure to 100 nM Tat.

### Permeability assay

The paracellular permeability of RPE cells was determined by measuring the apical-to-basolateral movement of sodium fluorescein (MW: 376 Da), using a slightly modified version of the technique of Hartnett et al [23]. Briefly, to assess the fluid flux across the monolayer, sodium fluorescein mixed in DMEM (25 mg/ml sodium fluorescein) was added to the apical compartment of the inserts after the indicated treatment. 100 μl of fluid was collected from the basolateral compartment of each filter at 20, 40 and 60 min after adding sodium fluorescein, and then transported to 96-well black culture plates (Corning Costar, Cambridge, MA) to measure the fluorescence. The same volume of the appropriate medium was added to replace the medium removed. The fluorescence was measured by a multifunctional microplate reader (emission: 525 nm, excitation: 440 nm). The basolateral-to-total fluorescence ratio was determined for each group, and expressed as a percentage, with larger percentage indicating greater permeability. The fluorescence of DMEM mixed with 25 mg/ml sodium fluorescein was taken as the total fluorescence.

### Real-time reverse-transcriptase polymerase chain reaction

Total RNA was isolated with TRIzol reagent. Real-time quantitative reverse-transcriptase polymerase chain reaction with SYBR (real-time qRT-PCR) was performed with Super-Script™ III Platinum1 Two-Step qRT-PCR kit (Invitrogen, Carlsbad, CA) on ABI PRISM 7000 sequence detection PCR system (Applied Biosystems, Foster City, CA) according to the manufacturer's protocol. Primers for human occludin, claudin-1, -2, -3, -4, and -5, and glyceraldehyde-3-phosphate dehydrogenase (GAPDH) were designed with Beacon Designer v 4.0 (Premier Biosoft, USA) (see Table 1 for the sequences). GAPDH was used as an internal control. The expression levels of occludin and claudin-1 to -4 are presented relative to those in the control group. To validate our real-time qRT-PCR protocol, melting-curve analysis was performed to check for the absence of primer dimers.

**Table 1:** Primer sequence used for real time RT-PCR

Gene	Accession number	upper	lower
Occludin	NM_002538	5' CATTGCCATCTTTGCCTGTG3'	5' AGCCATAACCATAGCCATAGC3'
Claudin1	NM_021101	5' CAGGCTACGACCGCAAC3'	5' CAGGCTACGCAAGGAC3'
Claudin2	NM_020384	5' CCCAAACCCACTAATCACATC3'	5' GCCACTGCTTCTCCTTCC3'
Claudin3	NM_001306	5' CAGGCTACGACCGCAAGGAC3'	5' GGTGGTGGTGGTGGTGTTGG3'
Claudin4	NM_001305	5' GGCGTGGTGTTCCTGTTG3'	5' AGCGGATTGTAGAAGTCTTGG3'
Claudin5	NM_003277	5' TACCGCAGGAAGAGGAGCAG3'	5' GCCCGAAGCAGCCAATCC3'
GAPDH	NM_002046	5' TCTCTGCTCCTCCTGTTC3'	5' CTCCGACCTTCACCTTCC3'

### Western blot analysis

Cells were lysed with 200 μl of ice-cold lysis buffer (50 mM HEPES, 5 mM EDTA, 100 mM NaCl, 1% Triton X-100; pH 4) in the presence of a protease inhibitor cocktail (Roche, Germany). Protein concentrations were determined with the BCA protein assay kit (Pierce, Rockford, IL). Protein samples (20 μg) were resolved on 10% SDS-PAGE gels and transferred onto a polyvinylidene difluoride (PVDF) membrane (Millipore, Bedford, MA) in a semi-dry system (Bio-Rad, Hercules, CA). The membranes were incubated with specific antibodies against occludin (1:500), claudin-1 (1:200), claudin-2 (1:100), claudin-3 (1:200), claudin-4 (1:100), and β-actin (1:500). β-actin was used as a loading control in experiments of cell-associated proteins. Chemiluminescence and visualized by exposure to X-ray films. Optical densities of the bands were scanned and quantified with the Gel Doc 2000 (Bio-Rad). Data were normalized against those of the corresponding β-actin, and results were expressed as percentages relative to controls.

To examine ERK activity, cells were extracted with lysis buffer containing phosphatase and protease inhibitors. Equal amounts of total proteins were boiled in sample buffer and separated by SDS-PAGE. After immunoblotting with an ERK phospho-specific antibody (1:100), immunoreactive bands were visualized as previously described.

### Immunofluorescence microscopy

Confluent D407 cells were exposed to 100 nM HIV-1 Tat for 24 hours; controls consisted of untreated cells and cells exposed to 100 nM heat-inactivated Tat for 24 hours. Controls and Tat-treated cells were washed with PBS, fixed for 30 min with 4% paraformaldehyde, permeabilized with 1% Triton-PBS (10 min at room temperature), and blocked with 2% BSA-PBS (1 hour at room temperature). Cells were then incubated with primary antibodies overnight at the following concentrations: anti-occludin (10 mg/ml), anti-claudin-1 (2 mg/ml), anti-claudin-2 (4 mg/ml), anti-claudin-3(4 mg/ml), anti-claudin-4(4 mg/ml). Cells were rinsed with 1% BSA-PBS and incubated for 1 hour with a fluorescein-conjugated secondary antibody (diluted 1:50 in 1% BSA-PBS). Cells were then rinsed three times with PBS, mounted in Vectashield medium, sealed, and analyzed by confocal microscopy (TCS NT, Leica). For occludin immunofluorescence, cells were preextracted according manufactuer's protocol before fixation and permeabilization.

### NF-κB DNA binding activity

Nuclear proteins were isolated by NE-PER^® ^Nuclear and Cytoplasmic Extraction Reagents according to the protocols supplied by the manufacturer. The DNA binding activity of NF-κB p50 and p65 subunits was assayed by NF-κB Transcription Factor Assay Chemiluminescent kit (Chemicon, Temecula, CA). Briefly, 2 μg nuclear extracts were incubated with the capture probes, double stranded bitinylated oligonucleotide containing the flanked DNA binding consensus sequence for NF-κB (5'-GGGACTTTCC-3'). The mixture was then transferred to a streptavidin-coated plate. The bound NF-κB transcription factor subunits p50 and p65 were detected with specific primary antibodies. A horseradish peroxidase-conjugated secondary antibody was then used for chemiluminescent detection. The relative light unit (RLU) values were measured using a LUMIstar Omega microplate reader (IMGEN, Washington ST).

### Statistical Analysis

Differences between groups were assessed by using one-way ANOVA with the SPSS 13.0 program (SPSS, Chicago, IL), with a probalility value of *P *< 0.05 considered indicative of statistically significance.

## Results

### MTT Cell Viability Studies

**Figure 1:**
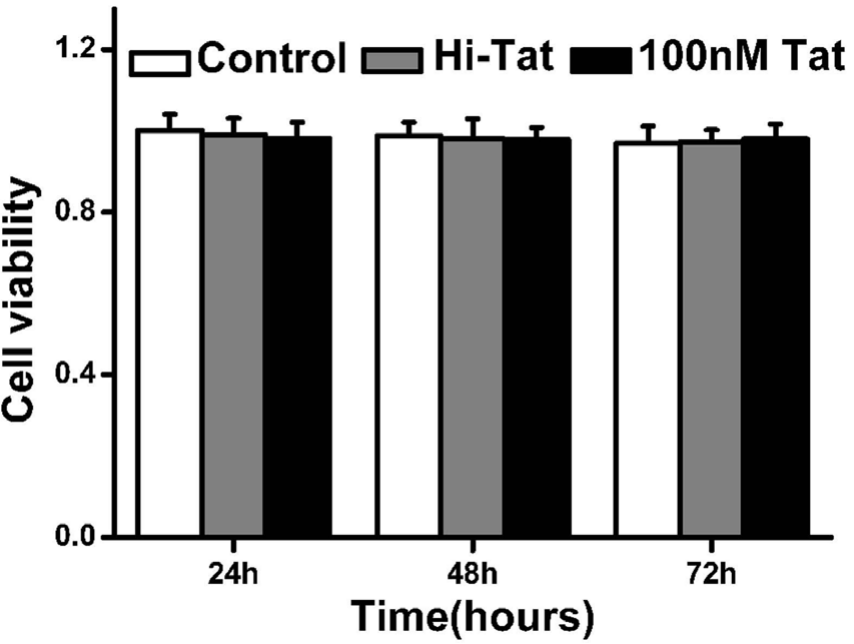
**Effect of 100 nM Tat on the viability of D407 cells**. The cells were incubated with 100 nM Tat and heat inactivated Tat for 24, 48, and 72 hours. Determination of the cell viability by the MTT assay indicated no significant differences between the groups (P > 0.05). Values shown represent the mean ± S.D. of four independent assays or experiments.

To exclude the possibility that changes in the barrier function resulted from cell death and the subsequent formation of holes in the monolayer, we tested the cytotoxic effects of 100 nM Tat on D407 cells. As shown in Figure 1, the average absorbance at 490 nm did not differ significantly between the control and treatment groups, indicating that the exposing cells to 100 nM Tat for 24~72 hours did not decrease cell viability relative to controls.

### HIV-1 Tat Induces destruction of barrier function in RPE

**Figure 2:**
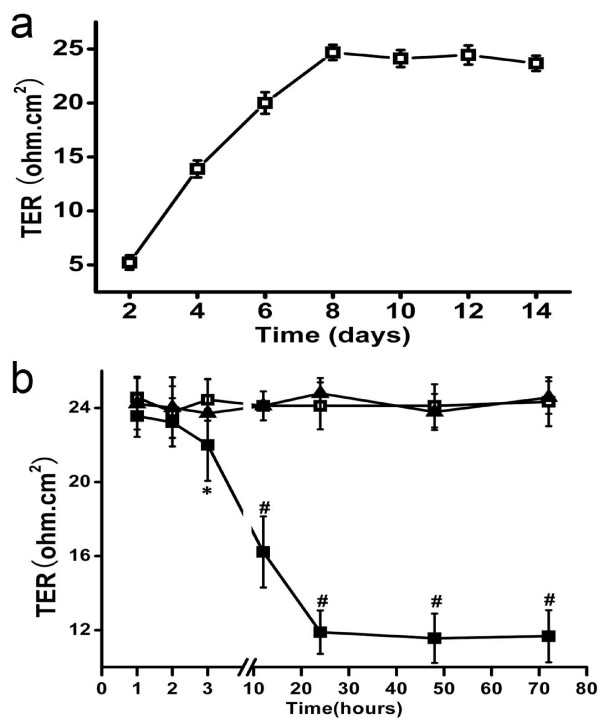
**Effect of Tat on the Transepithelial Electrical Resistance in D407 cells**. (a): From 2 days after seeding, the TER was measured every other day to monitor the time course of the TER of D407 cells. The TER gradually increased, peaking at day 8 and then remaining stable for 1 week. Values shown represent the mean ± S.D. of three independent assays or experiments. (b): The effects of 100 nM the Tat on TER of D407 cells were measured at 1, 2, 3, 12, 24, 48, and 72 hours after stimulation. The TER values were stable in the control group (□) and heat-inactivated group (▲) throughout the experiment, with no significant differences between them. However, treatment with 100 nM Tat(■) induced a significant decrease in the TER beginning at 3 hours, with a further decrease at 12 hours and a maximum effect at 24 hours, which was maintained to 72 hours. Values shown represent the mean ± S.D. of three independent assays or experiments. (* *P *< 0.05, # *P *< 0.01).

The TER appeared to be somewhat affected by the serum, so we reduce the serum concentration of the medium to 1% from day 3 when cells reached confluence, and measured the TER every other day. The TER of D407 cells gradually increased on the subsequent days, peaking at day 8 and then remaining stable for 1 week (Figure 2a). Mennel [24] suggested that obtaining stable values on 2 subsequent days indicated the formation of a tightly coupled cell monolayer, and hence we decided to begin treating the cells with 100 nM Tat from day 10.

The TER of D407 cells was measured at 1, 2, 3, 12, 24, 48, and 72 hours after treatment with 100 nM Tat. A reduction in the TER was first evident after 3 hours of treatment (*P *< 0.05). Continuous culturing of cells for longer periods further reduced the TER, with a maximum effect after 24 hours of treatment (*P *< 0.01) that was maintained to 72 hours. The TER of control groups (untreated and treated with Hi-Tat) remained unchanged throughout the experiment. (Figure 2b)

**Figure 3:**
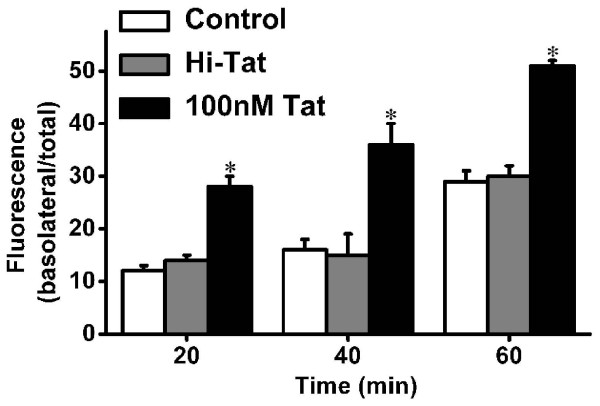
**Effect of Tat on RPE paracellular permeability**. Vertical axis shows the basolateral-to-fluorescence ratio and the horizontal axis shows the time after the addition of the molecule. Data are mean and S.D. Values shown represent the mean ± S.D. of three independent assays or experiments. Control: D407 cells incubated with standard medium; 100 nM Tat: D407 cells treated with medium containing 100 nM HIV-1 Tat protein for 24 hours. (*P < 0.05).

The permeability to sodium fluorescein, which has a low molecular weight, is regarded as a reliable marker of paracellular permeation. The permeability values of cells as measured at 20, 40, and 60 min after treatment with 100 nM Tat for 24 hours were all significantly higher than those of cells in the standard medium and the Hi-Tat contained medium, indicating that treating D407 cells with 100 nM Tat for 24 hours induced a loss of junctional integrity (Figure 3).

### HIV-1 Tat Induces Genes and Proteins Expression of TJs in RPE

The real-time quantitative reverse-transcriptase polymerase chain reaction demonstrated that occludin and claudin-1 to -4 were expressed in D407 cells, whereas there was no expression of claudin-5, similar to those from studies on claudins in another RPE cell line ARPE19 [25].

**Figure 4:**
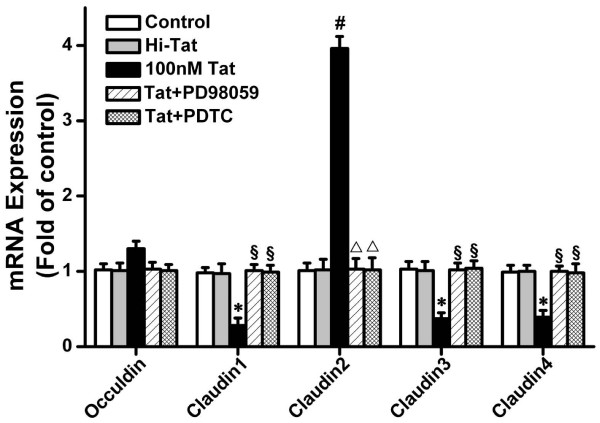
**Effect of Tat on the mRNA expression of tight junction proteins**. D407 were treated in 100 nM HIV-1 Tat for 24 h with or without pretreatment with the NF-κB inhibitor PDTC (100 μM) and the ERK inhibitor PD98059 (30 μM) for 1.5 h. The relative expression level of each gene is expressed as fold induction compared with control group. Data are mean and S.D. Values shown represent the mean ± S.D. of three independent assays or experiments. The genes examined are shown on the horizontal-axis. (*P < 0.05, ^#^*P *< 0.01 vs. control; ^§^*P *< 0.05, ^Δ^*P *< 0.01 vs. HIV-1 Tat protein alone).

The expressions of claudin-1, -3, and -4 genes were downregulated in D407 cells treated with 100 nM Tat, whereas that of the claudin-2 gene was upregulated. However, the expression of the occludin gene did not differ between cells treated with 100 nM Tat and control cells. (Figure 4)

**Figure 5:**
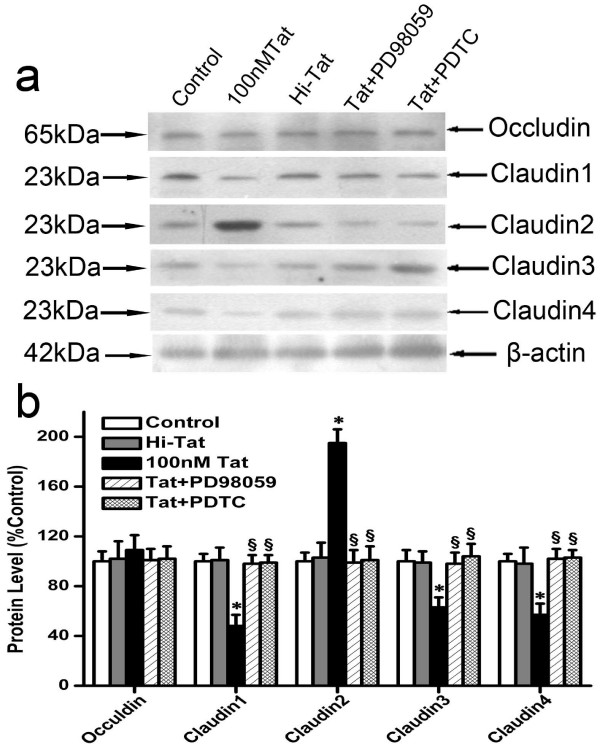
**Effect of Tat on the expression of tight junction proteins**. (a): D407 were treated in 100 nM HIV-1 Tat for 24 h with or without pretreatment with the PDTC (100 μM) and PD98059 (30 μM) for 1.5 h. Equal loading of protein was monitored using a specific antibody to β-actin.(b): Results of scanning densitometry of the exposed films. The relative expression level of each protein is expressed as percentage compared with control group. Values shown represent the mean ± S.D. of three independent assays or experiments. The proteins examined are shown on the horizontal-axis. (*P < 0.05 vs. control; ^§^*P *< 0.05 vs. HIV-1 Tat protein alone).

Bands were evident at approximately 65 and 23-kDa for occludin and claudins(1–4), respectively (Figure 5a). Consistent with the qRT-PCR observations, Tat (100 nM) reduced the expression of claudin-1, -3, and -4, increased that of the claudin-2, and had no effect on that of the occludin (Figure 5b).

**Figure 6:**
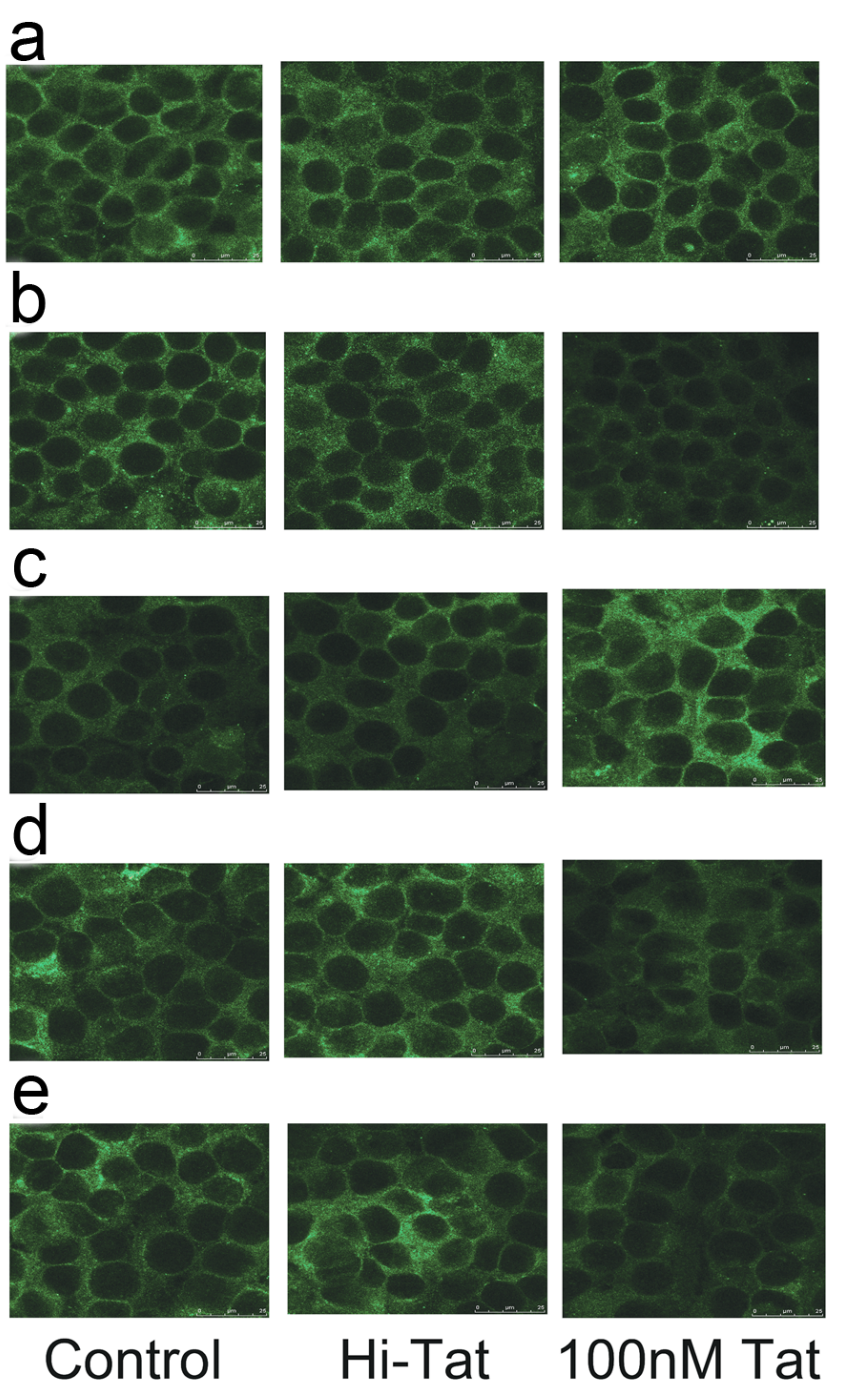
**Immunofluorescent staining of tight junction proteins in RPE**. D407 were treated with 100 nM HIV-1 Tat and Hi-Tat for 24 h. Occludin and claudin-1 to -4 expressions were determined by immunofluorescence microscopy. (a) Occludin, (b) claudin-1, (c) claudin-2, (d) claudin-3, and (e) claudin-4. Bar, 25 μm.

The results of immunofluorescence microscopy are shown in Figure 6. Junctional staining of each peptide was observed both in control cells and in cultures treated with Hi-Tat and 100 nM Tat. As for the qRT-PCR and Western blotting data, 100 nM Tat reduced the amount of staining of claudin-1, -3, and -4 (Figure 6a, b, c), increased that of claudin-2 (Figure 6d), and had no effect on the staining pattern of occludin (Figure 6e).

### HIV-1 Tat Induces ERK Phosphorylation and NF-κB DNA binding activity in RPE

**Figure 7:**
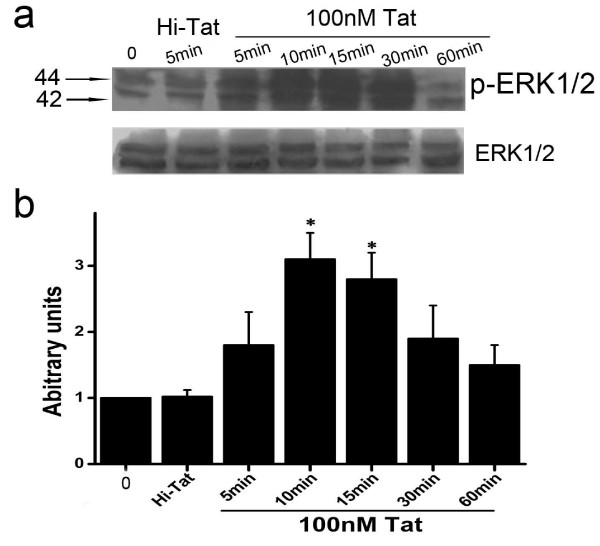
**Effects of HIV-1 Tat on ERK Phosphorylation in RPE**. (a): ERK1/2 phosphorylation was determined using an antibody specific to phospho-ERK1/2 after incubation of D407 with 100 nM HIV -1 Tat and Hi-Tat for various times, as indicated. Equal loading of protein was monitored using a specific antibody to total ERK. (b): Results of scanning densitometry of the exposed films. Data are expressed as arbitrary units of intensity relative to the control value, and are the mean ± S.D. of 3 independent experiments (*P < 0.05).

To determine the intracellular pathways that participate in changes in RPE induced by HIV-1 Tat, we examined whether the phosphorylation of ERK was induced in our cellular models upon treatment with HIV-1 Tat. D407 cells, starved for 24 hours in serum-free medium, were stimulated with 100 nM Tat for different time durations. As shown in Figure 7, 100 nM Tat was able to induce a large increase in ERK1/2 phosphorylation levels after 5 min of culture. The ERK1/2 activation levels remained at the same levels for 15 min, and began to decrease at 30 min.

**Figure 8:**
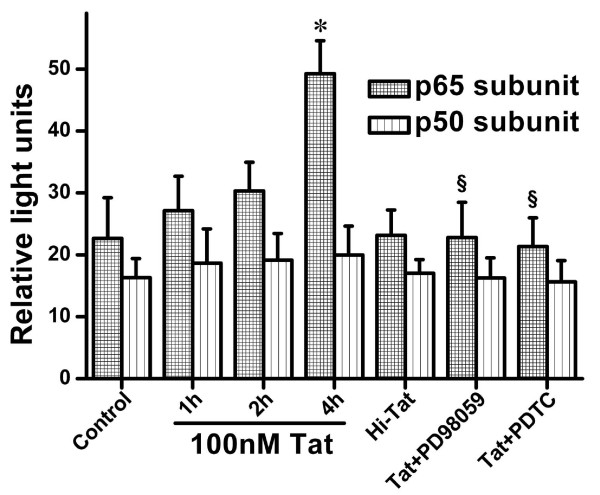
**Effects of HIV-1 Tat on NF-κB DNA binding activity in in RPE**. D407 were treated with HIV-1 Tat protein (100 nM) for 1, 2, and 4 h. Nuclear extracts were prepared. DNA binding activity was determined using 2 μg of nuclear protein as described in MATERIALS AND METHODS. To determine the effects of PDTC and SB-203580 on HIV-1 Tat protein induced NF-κB activation in RPE, D407 were treated in 100 nM HIV-1 Tat and Hi-Tat for 4 h with or without pretreatment with the PDTC (100 μM) and PD98059 (30 μM) for 1.5 h. Values shown represent the mean ± S.D. of three independent assays or experiments (*P < 0.05 vs. control; ^§ ^*P *< 0.05 vs. HIV-1 Tat protein alone).

Thereafter, we investigated whether the NF-κB transcriptional activity was associated with the effects induced by HIV-1 Tat protein, we examined NF-κB DNA binding activity after exposing D407 to 100 nM Tat for 1, 2, and 4 h. It was clearly shown that HIV-1 Tat protein significantly induced NF-κB DNA binding activity compared with control in a time-dependent fashion. The analysis of RLU showed that NF-κB p65 DNA binding activity induced by HIV-1 Tat protein at 4 h was significantly increased compared with the controls. In contrast, no significant difference was observed for the activation of the p50 subunit (Figure 8).

### PD98059 and PDTC Inhibit the Destruction of Barrier and Expression of TJs in RPE Induced by HIV-1 Tat

**Figure 9:**
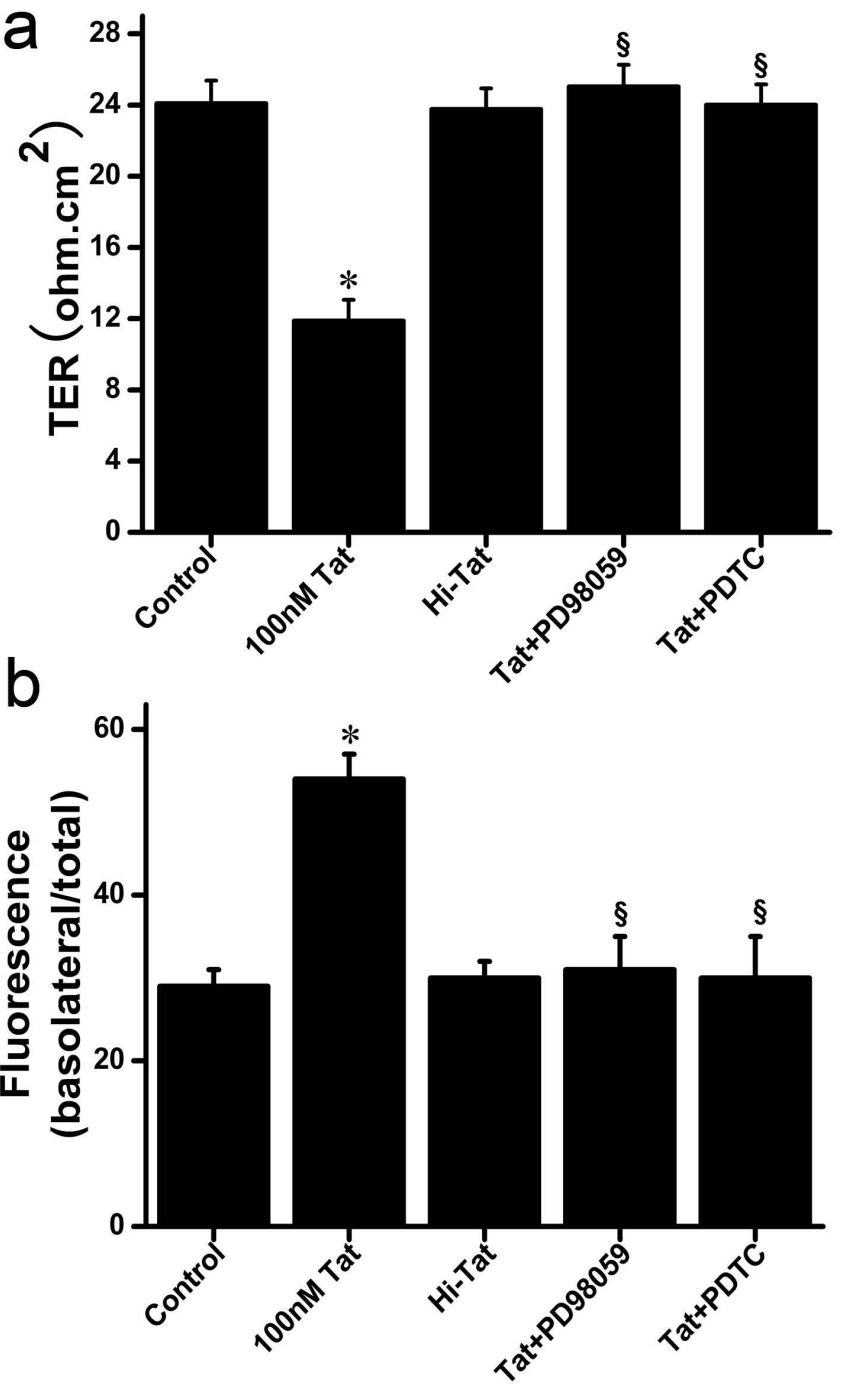
**Effects of PDTC and PD98059 on HIV-1 Tat induce destruction of barrier in RPE**. D407 were treated in 100 nM HIV-1 Tat and Hi-Tat for 24 h with or without pretreatment with the PDTC (100 μM) and PD98059 (30 μM) for 1.5 h. The TER and paracellular permeability were determined as previously described. Data are the mean ± S.D. of 3 independent experiments (*P < 0.05 vs. control; ^§ ^*P *< 0.05 vs. HIV-1 Tat protein alone).

To confirm whether the ERK1/2 and NF-κB activation was involved in the destruction of the barrier and expression of TJs in RPE induced by HIV-1 Tat protein, we pretreated D407 with the ERK specific inhibitor PD98059 and NF-κB inhibitor PDTC before stimulation with HIV-1 Tat protein. D407 cells were incubated with PD98059 (30 μM) or PDTC (100 μM) for 1.5 h and then were treated with HIV-1 Tat protein (100 nM) for 24 hours. The changes in barrier function and expression of TJs were detected as previously described. The results showed that both PDTC and PD98059 pretreatment abrogated the destruction of barrier and expression of TJs in RPE by HIV-1 Tat protein compared with HIV-1 Tat protein-alone (Figure 4, 5, 9). These data further suggest that both NF-κB and p38 MAPK may be involved in the regulation of HIV-1 Tat protein-induced biological effects.

### PDTC and PD98059 Inhibit NF-κB DNA Binding Activity Induced by HIV-1 Tat

To determine the relationship between NF-κB and ERK MAPK pathways in the regulation of HIV-1 Tat protein-induced effects, we used PDTC (100 μM) and PD98059 (30 μM) to pretreat the D407 for 1.5 h and then exposed the cells to 100 nM HIV-1 Tat for 4 h. The results showed that PDTC and PD98059 pretreatment noticeably decreased HIV-1 Tat-induced NF-κB DNA binding activity compared with HIV-1 Tat protein treatment alone (Figure 8).

## Discussion

Ocular manifestations are common in patients with AIDS. Many HIV patients suffer from decreased visual acuity, which may severely affect the quality of their lives. The mechanisms of HIV-1 entry into the eyes and the subsequent destruction of the homeostasis of the intraocular microenvironment remain obscure. Since most published research about the retina of HIV patients has focused on opportunistic infections and the resulting retinitis [26], few studies have investigated the direct effects of RPE.

There is increasing evidences of multifunctional effects of Tat that depends on the cell type and the degree of cellular maturation. We postulated that HIV-1 Tat protein could alter the expression of specific tight-junction proteins and disturb the blood retinal barrier, and contributes to HIV trafficking into the eyes.

The D407 is a spontaneously arising RPE cell line, which retains many of the metabolic and morphologic characteristics of RPE cells in vivo [27,28]. D407 cells possess intercellular junctional complexes, and have been used to model the oBRB [29]. We therefore used D407 cells in the present study to test the above-mentioned hypothesis.

The results from our experiments indicate that treatment with 100 nM Tat, which does not cause the cell death, disturbs the barrier function of the oBRB. In the presence of AIDS, HIV-1 Tat arriving at the choroidal capillary bed, can interact with the RPE and destroy the barrier function of oBRB. Because the choroid vasculature is fenestrated and abundant in blood, the destruction of oBRB would expose the retina to immune cells such as monocytes, macrophages, and dendritic cells. We therefore suppose that HIV trafficking into the eyes is also mediated through a "Trojan horse" mechanism, in which HIV-infected circulating monocytes enter the eyes through breaches of the oBRB, as in the brain and BBB [21].

It has been verified that anomalies in the expression and distribution of occludin and claudins are responsible for the occurrence and development of many disease. Claudins are localized to the site of close membrane apposition within TJs. They are detected in both epithelial and endothelial cells in all tissues that contain TJs, and form a complex with occludin and junctional adhesion molecules [30,31]. In the present study, HIV-1 Tat-induced decreases in expressions of claudin-1, 3, 4 and significant increases in claudin-2 were detected in D407 cells. They were all consistent with the decrease in the TER and the increased permeability. Although we failed to show the changes in occludin expression in the present study, we also found the decreases in expression of ZO-1 in another experiment (data not shown). Moreover the Tat-related mRNA and protein variation of claudins is relatively low, so we cannot exclude the possibility that other junctional proteins are also modulated by Tat and contribute to the observed effects on barrier function. The relationship between TJs and the oBRB during HIV infection still need to be elucidated.

It was reported that Tat can induce oxidative stress and excitotoxicity in the RPE and brain endothelial cells [22,32], indicating that oxidative stress plays a major role in the HIV-1 Tat-mediated retinal dysfunction associated with AIDS retinopathy. H_2_O_2 _was shown to influence the expression of TJs in cultured RPE in a similar fashion as HIV-1 Tat (unpublished data). Numerous studies have suggested that HIV-1 Tat can trigger activation of redox-regulated cell signaling pathways, of which ERK MAPK could alter the composition of claudins within the TJ complex and change TJ permeability rapidly [33-35]. We further determined whether these pathways are involved in the regulation of claudins expression that was observed in the present study. Our study's results have shown clearly that the activation of ERK1/2 is important for the destruction of barrier and expression of TJs in HIV-1 Tat treated RPE. First, HIV-1 Tat has induced the phosphorylation of ERK1/2. Second, PD98059, a specific inhibitor of MEK-ERK inhibited HIV-1 Tat-induced changes in barrier and expression of TJs. But as the ERK1/2 activation kinetics were not studied in untreated control cells, the global effects of HIV-1 Tat on ERK1/2 activation dynamics in RPE are difficult to compare.

NF-κB is one of the transcription factors that may be controlled by the redox status of the cells [36]. Activation of NF-κB is controlled by a family of inhibitors. Upon stimulation, after the active complex p65/p50 of NF-κB is released from the inhibitor, and translocate from the cytoplasm to the nucleus, where they bind target genes and stimulate transcription. Although exogenous HIV-1 Tat protein is known to activate NF-κB in immune cells and endothelial cells, it is not well known whether exogenous HIV-1 Tat protein is able to activate the NF-κB pathway in epithelial cells [37]. The results showed an increase in NF-κB DNA binding activity in nuclear extracts from HIV-1 Tat treated RPE. The specific NF-κB inhibitor, PDTC, also inhibited the changes in barrier function, expression of TJs, and the activation of NF-κB induced by HIV-1 Tat. These indicated that the effects of HIV-1 Tat on barrier function of RPE were NF-κB dependent.

Our study's results showed that both NF-κB and ERK1/2 MAPK were involved in the effects of HIV-1 Tat on the barrier function of RPE. Generally, NF-κB is not thought to be a transcription factor activated by ERK MAPK [38]. However, several reports indicate that ERK MAPK is also an important activator of NF-κB [39,40]. Our study particularly shows that the NF-κB DNA binding activity induced by HIV-1 Tat was abolished by the PD98059, a specific inhibitor of ERK. This implies that NF-κB acts as a downstream substrate of ERK MAPK during barrier destruction in RPE induced by HIV-1 Tat.

## Conclusion

The present study is the first to provide evidence that HIV-1 Tat induced changes in the claudin composition of TJs, thereby, contributing to the destruction of the barrier function of the RPE and eventually inducing the pathogenesis of HIV-related ocular diseases. The effects of HIV-1 Tat on the barrier function of the RPE may be mediated by ERK MAPK and NF-κB activation, which may represent potential targets for novel therapeutic approaches for the retinopathy induced by HIV infection. But it still needs to be confirmed in human primary RPE cells or *in vivo *situation.

## Abbreviations

RPE: retinal pigment epithelial cell; TER: transepithelial electrical resistance; TJs: tight junctions; oBRB: outer blood-retina barrier; DMEM: Dulbecco's modified Eagle's medium; FBS: fetal bovine serum; PBS: phosphate-buffered saline; BSA: bovine serum albumin; DMSO: dimethyl sulfoxide; qRT-PCR: quantitative reverse-transcriptase polymerase chain reaction; PVDF: polyvinylidene difluoride; MAPK: mitogen activated protein kinase; ERK: extracellular signal-regulated kinases; NF-κB: nuclear factor kappaB; PDTC: pyrrolidine dithiocarbamate.

## Competing interests

The authors declare that they have no competing interests.

## Authors' contributions

Conceived and designed the experiments: ZZ, HZ. Performed the experiments: LB, XL, QY, HL. Analyzed the data: LB, WY. Contributed reagents/materials/analysis tools: HZ. Wrote the paper: LB, HZ.

## Pre-publication history

The pre-publication history for this paper can be accessed here:


